# In-Plane and Out-of-Plane Investigation of Resonant Tunneling Polaritons in Metal–Dielectric–Metal Cavities

**DOI:** 10.1021/acs.nanolett.2c04864

**Published:** 2023-02-06

**Authors:** Aniket Patra, Vincenzo Caligiuri, Bruno Zappone, Roman Krahne, Antonio De Luca

**Affiliations:** † Dipartimento di Fisica, Università della Calabria, via P. Bucci 33b, 87036 Rende CS, Italy; ‡ Consiglio Nazionale delle Ricerche−Istituto di Nanotecnologia (CNR-Nanotec), via P. Bucci 33c, 87036 Rende, Italy; § Optoelectronics Research Line, Istituto Italiano di Tecnologia, via Morego 30, 16163 Genova, Italy

**Keywords:** epsilon-near-zero, polaritons, strong coupling, surface forces apparatus, metal-dielectric multilayers

## Abstract

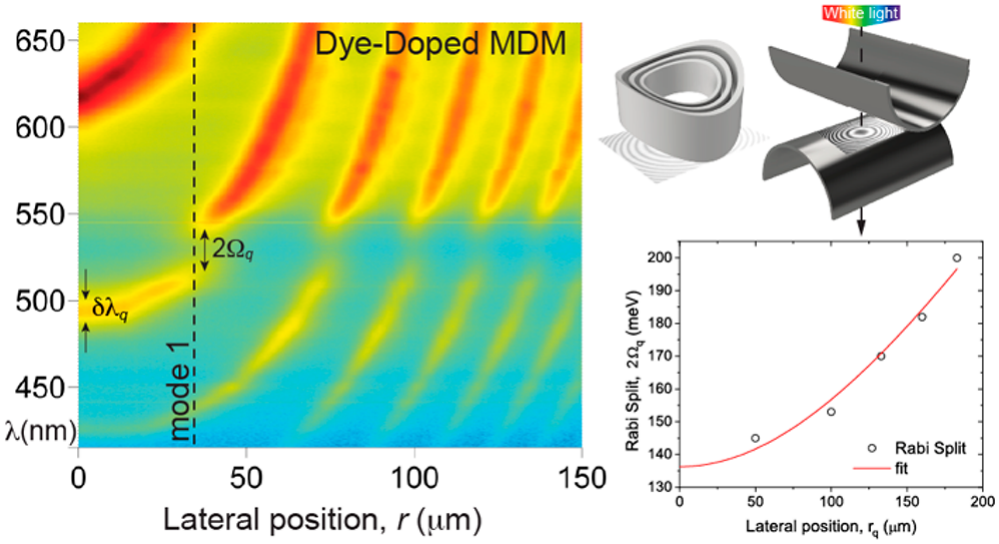

Polaritons can be generated by tuning the optical transitions of a light emitter to the resonances of a photonic cavity. We show that a dye-doped cavity generates resonant tunneling polaritons with Epsilon-Near-Zero (ENZ) effective permittivity. We studied the polariton spectral dispersion in dye-doped metal-dielectric-metal (MDM) cavities as a function of the in-plane (*k*_||_) and out-of-plane (*k*_⊥_) components of the incident wavevector. The dependence on *k*_||_ was investigated through ellipsometry, revealing the ENZ modes. The *k*_⊥_ dependence was measured by varying the cavity thickness under normal incidence using a Surface Force Apparatus (SFA). Both methods revealed a large Rabi splitting well exceeding 100 meV. The SFA-based investigation highlighted the collective nature of strong coupling by producing a splitting proportional to the square root of the involved photons. This study demonstrates the possibility of generating ENZ polaritons and introduces the SFA as a powerful tool for the characterization of strong light–matter interactions.

Light–matter interaction between a quantum system (light-emitting molecules, quantum dots, nanocrystals, etc.) and an electromagnetic resonator can be so strong that hybrid quasi-particles called polaritons are generated.^1−6^ Several systems have been proposed as resonators but, among them, Fabry–Perot resonators are the most broadly used.^7−19^ In a Fabry–Perot resonator with a single cavity, the resonances can be finely tuned by changing (i) the thickness *t*_D_ of the dielectric(s) in the cavity, (ii) the refractive index *n*_D_of the dielectric layer(s), (iii) the incidence angle θ_*i*_ of the input radiation, and (iv) its polarization^20^ (see schematic illustration in Section 1 in the Supporting Information (SI)).

In a planar Fabry–Perot cavity, monochromatic plane waves satisfy the dispersion relation *k*_||_^2^ + *k*_⊥_^2^ = (ω/*c*)^2^ε, where *k*_||_ = *k*_*i*_ sin(θ_*i*_) and *k*_⊥_ = *k*_*i*_ cos(θ_*i*_) are the in-plane and out-of-plane components of the incident wavevector (respectively parallel and normal to the layers), ε is the dielectric permittivity, ω is the frequency, and *c* is the speed of light. A resonance occurs when eq 1 is satisfied:^21,22^
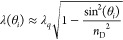
1

The resonant wavelength under normal incidence (*λ*_*q*_) can be calculated from the equation
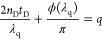
2where *q* is the modal order (*q* = 1, 2, 3, ...) and ϕ is the phase shift under reflection at the dielectric/metal interface.

Combining eq 2 with eq 1 shows that the same resonance wavelength can be obtained by either varying the incidence angle θ_*i*_ with a fixed cavity thickness *t*_D_, or varying *t*_D_ under normal incidence (θ_*i*_ = 0) (Section 1 in the SI).

It has been recently demonstrated that the resonances of a metal–dielectric–metal (MDM) resonator possess an epsilon-near-zero (ENZ) character, with a vanishing real part and a small imaginary part of the effective dielectric permittivity.^23^ Since polaritons inherit properties from both the “matter” (emitter) and the “photon” (cavity mode) counterparts, it is plausible to expect that they could inherit the ENZ features of the MDM resonances.

In this Letter, we report on the spectral dispersion of MDM cavities made of Ag as metal and a PVP-Rhodamine 6G (R6G) blend as the dielectric (sketch in Figure 1b), and we experimentally demonstrate that the polaritons inherit the ENZ character of the cavity modes. Spectroscopic ellipsometry was used to determine the in-plane dispersion as a function of *k*_||_ (i.e., with respect to the incidence angle θ_*i*_), whereas the out-of-plane dispersion was determined as a function of the cavity thickness *t*_D_ under normal incidence. The latter measurement were performed with a surface forces apparatus (SFA), which is an instrument originally designed to quantify surface forces at the nanoscale, and was recently adapted to vary the dielectric thickness of MDM cavities from the micrometer scale down to a few nanometers.^24^ The SFA experiments highlighted the collective nature of strong coupling as a dependence of the Rabi splitting on the number of photons in the cavity, an effect that is often overshadowed by the dependence on the number of emitters.

**Figure 1:**
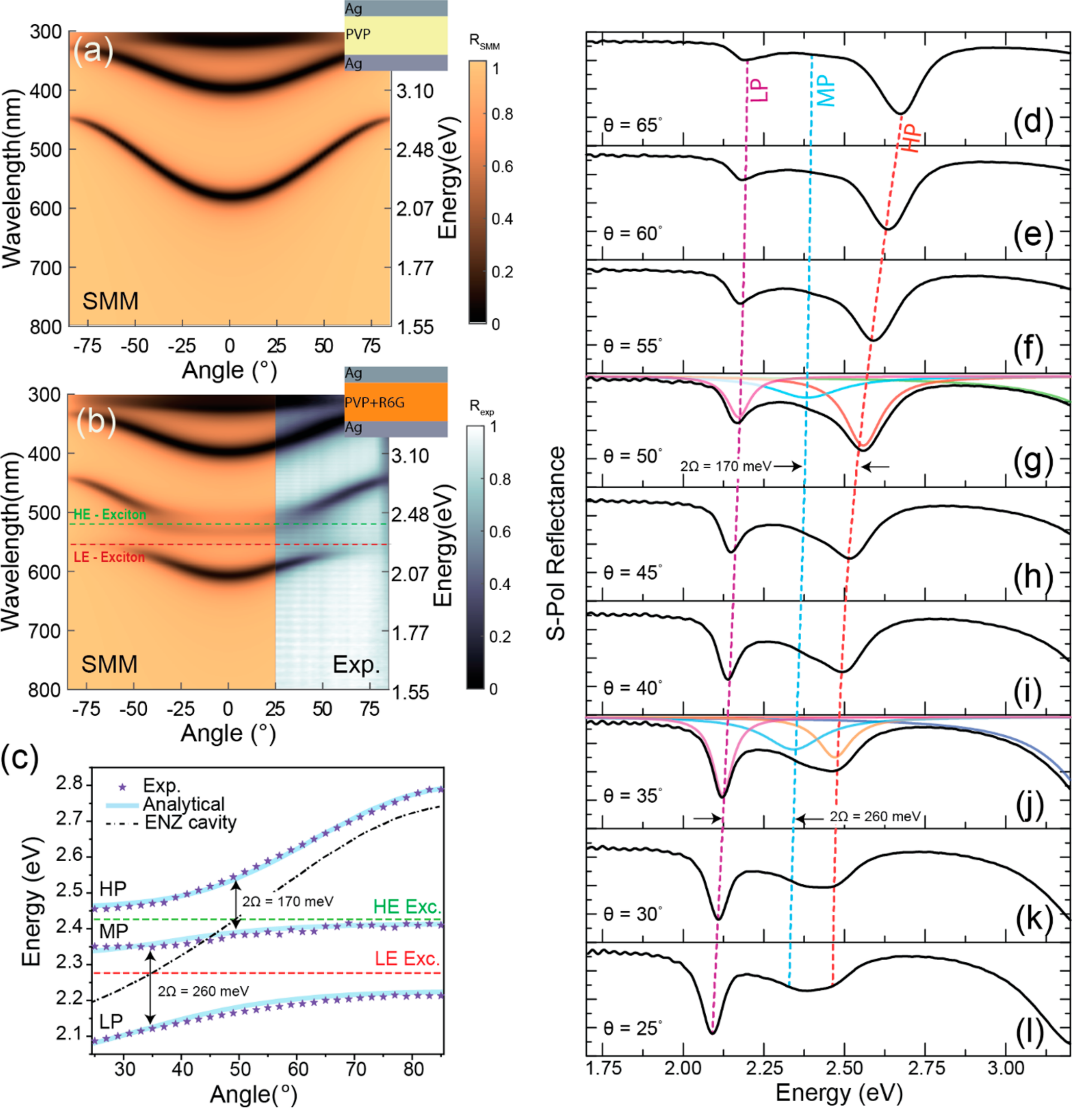
(a) Reflectance of an undoped MDM cavity (see inset) calculated with the SMM for *s*-polarization, and PVP and Ag layers with thicknesses of 350 and 35 nm, respectively. (b) Experimental and calculated *s*-polarization reflectance of the doped MDM cavity with R6G embedded in the PVP layer (see inset). (c) Analytically calculated (solid blue lines) and experimentally measured (blue stars) angular dispersion of resonances in the doped MDM cavity. Hybridization of the MDM cavity mode (black dash-dotted curve) with the LE and HE excitons of R6G (dashed horizontal lines) generates the low, middle, and high polariton branches (LP, MP, and HP), respectively. (d–l) Experimental reflectance at different incident angles θ_*i*_.

We first consider the interaction between the optical transitions of R6G molecules and ENZ cavity modes as a function of the parallel wavevector component *k*_*||*_. The angular dispersion of the MDM cavity is characterized by a blue-shift of the resonance wavelength as the angle of incidence θ_*i*_ is increased (eq 1 and Figure 1a).^22^ Note that varying the angle of incidence provides the same information that can be obtained via *k*-space spectroscopy.

To highlight the effect of optical coupling, we calculated the angle-dependent reflectance of an undoped (i.e., dye-free) Ag/PVP/Ag MDM using Scattering Matrix Method (SMM) simulations, and we compared the results with our experiments obtained for a R6G-doped cavity with the same geometry (PVP and Ag layers with thicknesses of 35 and 315 nm, respectively; see Figures 1a and 1b). We consider the reflectance for the *s*-polarization. When the incidence angle increases, the resonance modes of the undoped cavity blue-shift (see Figure 1a).

Rhodamine 6G hosts two main active optical transitions peaked at wavelengths of 508 and 546 nm, which translate into a high-energy (HE) and a low-energy (LE) excitonic absorption peak, respectively (see Section 2 of the SI).^25^ The HE exciton has an energy of ∼2.441 eV that can be related to vibronic sidebands.^25^ It is characterized by a small transition dipole moment, as confirmed by the low oscillator amplitude in the Gaussian fit of the imaginary part of the refractive index of dye-doped PVP + Rhodamine 6G layer measured via spectroscopic ellipsometry (see Section 2 of the SI). The LE exciton, on the other hand, occurs at an energy of ∼2.27 eV and possesses a large transition dipole moment associated with *S*_1_ π–π* bonding.^25^ Strong coupling between a single-transition “R6G-like” molecule and a cavity mode has been recently investigated theoretically by Gallego et al.^26^ The authors considered rovibrational degrees of freedom and showed that dark hybrid states can be brightened in the strong coupling regime, leading to additional bright polaritonic states. However, this is not our case, since the two excitonic transitions, LE and HE, are already present in the absorption spectrum of R6G molecules, and we address a scenario of three coupled oscillators, in which two of them are represented by the two excitons and the third one is the cavity mode.

Off-resonance, the angular dispersion of the doped MDM cavity is similar to that of the undoped cavity, and the resonance wavelength blue-shifts as the incidence angle increases (see the band between 300 and 400 nm in Figure 1a). Near and on-resonance, when the cavity photon energy is close to an R6G transition, exciton-cavity polaritons are generated, and the dispersion of the resonant modes splits into polaritonic branches (Figure 1b). The splitting measured in reflection with s-polarization was accurately reproduced by SMM calculations (for the p-polarization, see Section 3 of ther SI), and the refractive index of both the Ag and R6G layers used in these calculations was measured via spectroscopic ellipsometry.

Figures 1b and 1c depict the polariton dispersions with avoided-crossing points between cavity modes and the LE and HE excitons of R6G at two distinct incidence angles: θ_in_ = ±35° and θ_in_ = ±50°, respectively. Since the HE and LE excitons have similar energies, they can both couple to the same cavity mode. This interaction can be modeled considering three coupled oscillators, producing three polaritonic dispersion branches, labeled as low (LP), middle (MP), and high (HP) polariton, according to their energy (see Figure 1c). For the interaction between the cavity mode and the LE exciton with large dipole moment, we obtained a large Rabi splitting 2Ω_LE-ph_ = 260 meV, whereas for the HE mode with a small dipole, we found a smaller splitting of 2Ω_HE-ph_ = 170 meV (obtained at the avoided-crossing points by fitting the reflectance curves as the sum of three gaussians). The anticrossings between all these polaritonic branches can be appreciated in Figure 1d–l, showing the s-polarized reflectance spectra acquired at angles spanning from 25° to 65°. Several ways have been proposed to characterize strong coupling, but the most broadly accepted condition is that the vacuum Rabi splitting 2Ω must be larger than the dissipative broadening of both the exciton (γ_e_) and the cavity mode (γ_ph_),^27^ e.g., 2Ω > (γ_e_ + γ_ph_)/2. In our case, we have 2Ω_LE-ph_ = 260 meV, γ_LE_ = 112 meV, and γ_ph(θ=35°)_ = 75 meV for the coupling between the LE exciton and the cavity mode occurring at ∼35°, whereas values of 2Ω_HE-ph_ = 169 meV, γ_HE_ = 174 meV, and γ_ph(θ=50°)_ = 96 meV for the coupling between the HE exciton and the cavity mode are observed at ∼50°. In the first case, we find (γ_LE_ + γ_ph(θ=35°)_)/2 = 0.094 eV and, therefore, the condition 2Ω_LE-ph_ > (γ_LE_+γ_ph(θ=35°)_)/2 is satisfied, confirming the strong coupling regime. In the latter case, we find (γ_HE_+γ_ph(θ=50°)_)/2 = 135 meV, again validating the condition for strong coupling.

As mentioned above, resonances in an MDM cavity can be interpreted as ENZ modes in a homogenized description of the complex effective permittivity.^23^ This concept can be applied to the MDM multilayers studied in this work as well. We performed spectroscopic ellipsometry measurements and considered the R6G-doped MDM cavity as a homogenized material, and measured the pseudodielectric permittivity ε of the system as an effective material property. The real (ε′) and imaginary parts (ε′′) of ⟨ε⟩ are shown in Figures 2a and 2b, as solid black lines.

**Figure 2:**
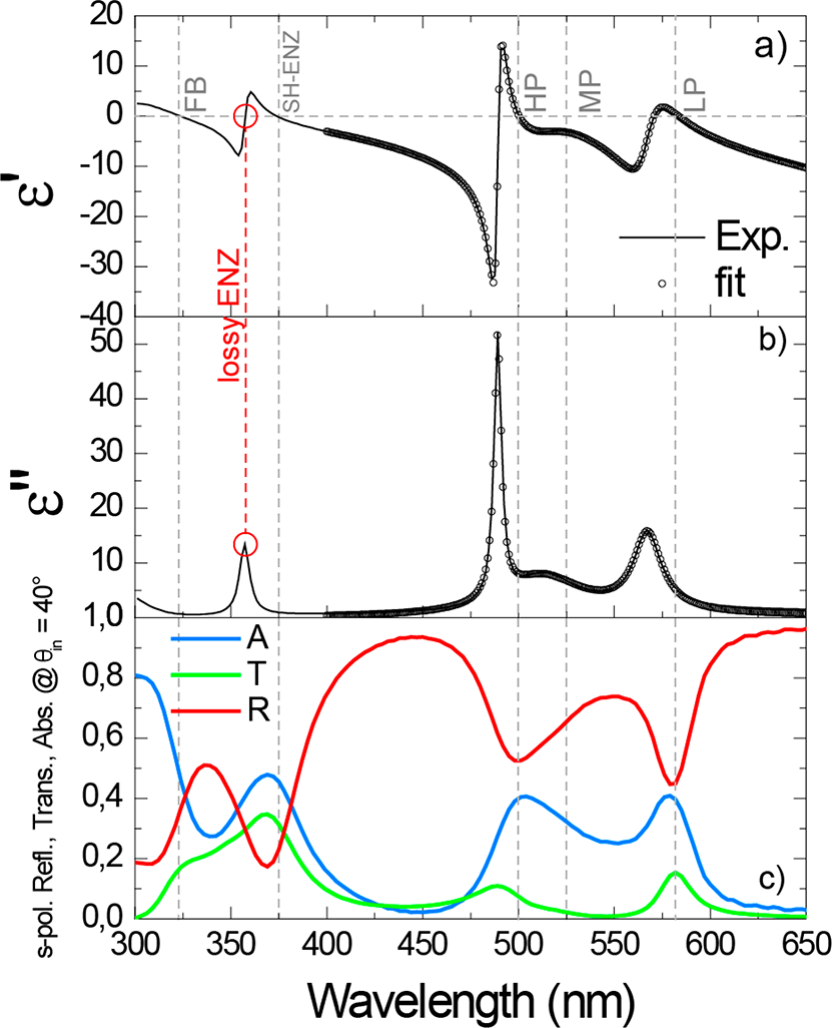
(a) Real part ε′ and (b) imaginary part ε″ of the experimentally measured pseudodielectric permittivity ⟨ε⟩ (solid black lines) of a R6G-doped MDM cavity, compared to the effective dielectric permittivity (black dots) analytically modeled through eq S2 in the SI. The measurement was done by ellipsometry at a 40° angle of incidence, the dispersion obtained from the analytic model is shown only within the polaritonic region, starting from 400 nm onward. (c) Experimentally measured reflectance *R* (red curve), transmittance *T* (green curve), and absorbance *A* (blue curve, obtained as *A* = 1 – *R* – *T*) for *s*-polarization. Low-loss ENZ modes correspond to transmittance (absorbance) peaks and reflectance dips with ε′ = 0 and small ε″. FB and SH-ENZ indicate the Ferrell–Berreman and second-harmonic ENZ mode, respectively. LP and HP are the lower and higher ENZ polaritons, respectively.

Several transitions with zero ε′ are present in Figure 2a. The one at 327 nm is the well-known Ferrell–Berreman mode of Ag (Figure 2a, labeled “FB”). The resonance labeled SH-ENZ is a pure cavity mode, whose ENZ features have been demonstrated in refs (23), (28), and (29). The red-circled zero-crossing labeled as “Lossy ENZ” corresponds to the central wavelength of the Lorentzian oscillator used to model the bare cavity mode and it is not of interest for our work, as well as all the zero crossings found in correspondence of the peaks of ε″ (high losses). The zero-crossing points labeled as HP and LP in Figure 2a correspond to polaritonic modes that manifest ENZ features. Crucially, in correspondence to these wavelengths, dips in reflectance (*R*, red curve of Figure 2c) are present together with peaks in transmittance (*T*, green curve of Figure 2c) and in absorbance (*A* = 1 – *R* – *T*, blue curve of Figure 2c). In contrast, in correspondence to MP, no zero crossing was found. This behavior can be explained by considering the Hopfield coefficients of the three polaritons (see Section 5 of the SI), which reveal that HP and LP manifest mainly a photonic nature, while MP has a marked excitonic, lossy character. The relation of the ENZ properties of the polaritons to their photonic or excitonic character are also supported by the low-loss spectral region of the HP and LP modes (PVP-R6G compound), with extinction coefficient κ < 0.02 (see the red curve in Figure S2c in the SI, calculated from the ellipsometric measurements of Ψ and Δ (Figures S2a and S2b)). Therefore, the HP and LP polariton branches inherit high-quality ENZ features from the cavity mode, and the real part of the effective permittivity crosses zero at the polariton frequencies (or wavelengths in Figure 2a). In contrast, polaritons belonging to the MP branch always lie within the lossy, high-absorbance energy range of R6G and do not manifest ENZ behavior.

At the HP and LP wavelengths, the ENZ permittivity shows low losses (Figure 2b), so that the effective polariton wavevector (*k* = *k*_0_ε_eff_^1/2^) almost completely vanishes, since ε_eff_ ≈ 0. This condition, which also sustains the propagation of polaritons generated in the dye-doped MDM cavity, is known as “resonant tunneling”.

To investigate the influence of the out-of-plane component of the incident wave vector on the polaritons, we used a SFA to measure the dispersion of MDM cavities as a function of a continuous variation of the cavity thickness *t*_D_. In this case, the cavity dielectric layer was a solution of PVP in ethanol, either pristine or doped with R6G molecules. The SFA technique is described in ref (30) and applications to multiple-beam interference and photonics can be found in ref (24). The fluid was confined between two cylindrical lenses with radius R = 2 cm, coated with a 30 nm Ag layer, facing each other at a distance *d* apart with their cylinder axes crossed at 90° (Figures 3a and 3b). The metal layers constituted the mirrors of a curved MDM cavity with a nonuniform thickness *t*_D_ of the dielectric fluid. Around the point of closest surface approach, the separation distance between the two cylindrical surfaces is given by eq 3:
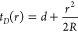
3where *r* is the lateral distance from the point of closet surface approach (*r* = 0) and *r* ≪ *R*. This geometry is equivalent to that of a sphere of radius *R* facing a plane at a distance *d* (see Figures 3a and 3b).

**Figure 3:**
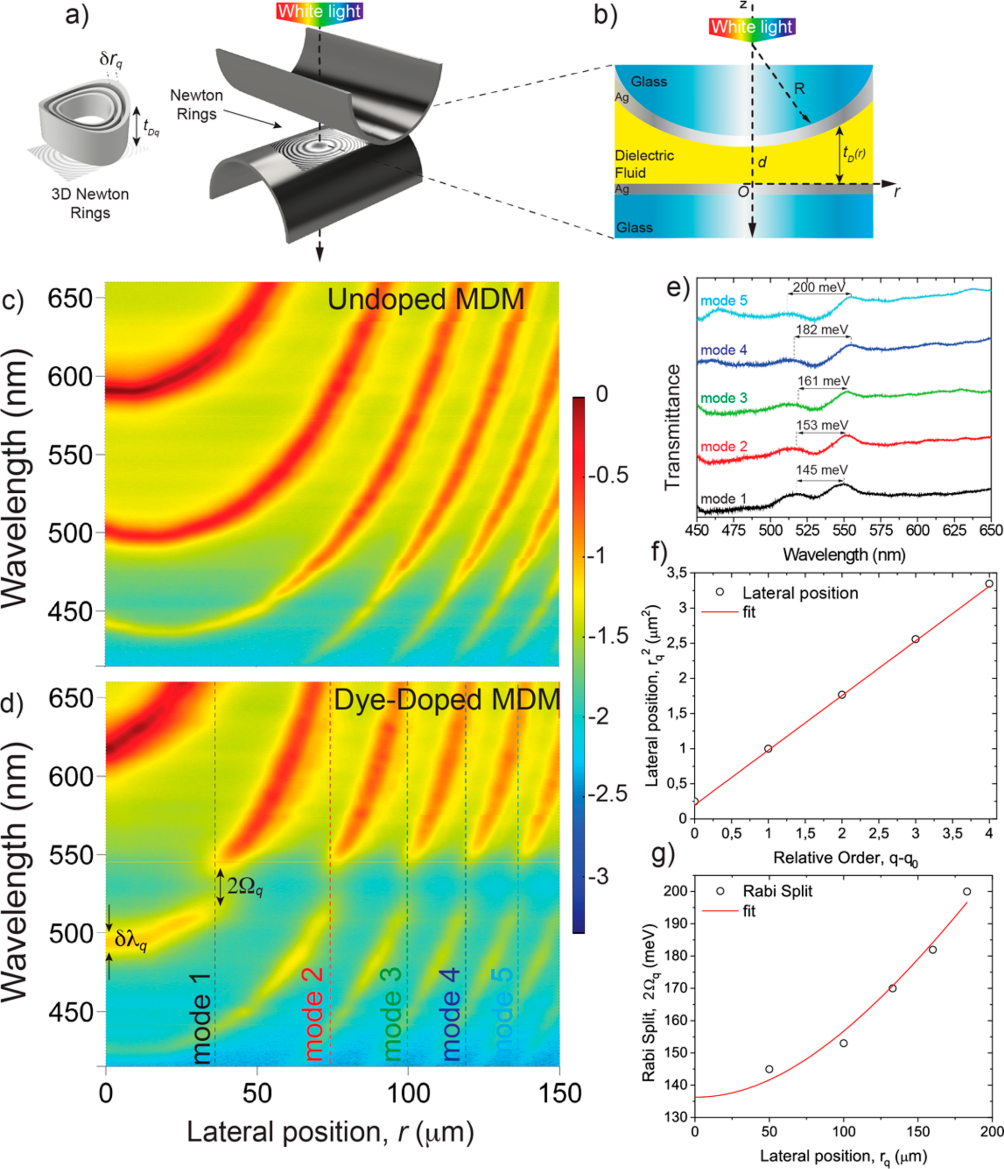
(a) 3D sketch of the SFA crossed-cylinder geometry highlighting the formation of the Newton rings. (b) Cross-section of the SFA geometry along the axis of the bottom cylinder. R = 2 cm is the cylinder radius, and *r* is the lateral distance from the point of closest surface approach, i.e., *r* = 0, where the surface separation distance is *d*. The Ag layers on the glass cylinders (thickness 30 nm) together with the dielectric fluid between them form a curved MDM cavity with nonuniform thickness *t*_D_. (c) Color plots showing the logarithm of transmitted intensity as a function of the wavelength λ and position *r* for a fixed distance *d* between the surfaces in a PVP-ethanol mixture (without R6G). (d) Similar color plot obtained for a dye-doped solution containing 1.5 wt % of R6G. (e) Transmittance spectra taken from panel (d), showing the Rabi splitting measured for five consecutive harmonics. (f) Linear increase of the squared lateral position r_*q*_^2^ measured from the spectra in panel (d), as a function of the relative order *q* – *q*_0_. (g) Parabolic increase of the Rabi splitting 2Ω_q_ as a function of the lateral position *r*_*q*_.

The light intensity *I* transmitted under normal incidence through the curved MDM cavity was collected using a microscope and directed with a right-angle prism into an imaging spectrograph coupled to a CCD camera. Therefore, the SFA setup is an ideal technique for directly revealing ENZ polaritons as it detects transmittance maxima corresponding to resonant tunneling cavity polaritons whose ENZ character was demonstrated above.^23^

This setup allowed to resolve *I* as a function of the wavelength λ and lateral position *r* over a field of view of ∼150 μm surrounding the point of closest surface approach (*r* = 0). Spectrograms *I*(*r*,λ) obtained for an undoped mixture of PVP and ethanol showed continuous resonance fringes with a parabolic shape reflecting the surface curvature (Figure 3c).

In eq 2, the phase shift ϕ due to reflection at the dielectric–metal interface varies slowly with the wavelength and, therefore, the resonance wavelength λ_*q*_ increases with *t*_D_ following an approximately linear dependence.^24^ Since *t*_D_ increases parabolically with *r* (eq 3), the resonance fringe of order *q*, i.e., λ_*q*_(*r*), presents a parabolic shape.

For R6G-doped PVP-ethanol solutions, the resonance fringes showed several anticrossings between the cavity modes (for different order *q*) and the RG6 excitons (∼510 nm). In Figure 3d, the splitting of up to five consecutive harmonics (high-order modes) were detected in the spectrograph field of view, demonstrating a rapid, single-shot measurement that captures the dispersion of several polariton modes.

To evaluate the coupling strength, we plotted the transmittance spectra at the five lateral positions *r*_*q*_, related to the mode of order *q*, corresponding to the different anticrossing points (Figure 3e) and measured the Rabi splitting 2Ω_*q*_ via Gaussian fits of the transmittance peaks (see Section 6 in the SI). The analysis shows that the splitting increases nonlinerarly with the mode radius *r*_*q*_ (Figure 3f). This finding can be interpreted by recalling the collective nature underlying the Rabi splitting. Indeed, according to the well-known Tavis–Cumming model, the Rabi splitting depends on (i) the numbers of photons *m*_*q*_, (ii) the number of the emitters *N*_*q*_, and (iii) the modal volume *V*_*q*_, as 2Ω_*q*_ ∝ [(*m*_*q*_ + 1)*N*_*q*_/*V*_*q*_]^1/2^. In most experiments, *N*_*q*_ is increased while *V*_*q*_ and *m*_*q*_ are fixed. In our case, instead, the ratio *c* = *N*_*q*_/*V*_*q*_ is constant, due to the uniform concentration of dye molecules in the polymeric solution. In contrast, the number of photons *m*_*q*_ increases with the lateral position *r*_*q*_ and, equivalently, with the cavity thickness *t*_*q*_ (see Section 7 of the SI). As a result, in the SFA geometry, the Rabi splitting scales as 2Ω_*q*_ = *At*_*q*_^1/2^ (where *A* is a constant), or equivalently 2Ω_*q*_ ∝ (*d + r*_*q*_^2^/2*R*)^1/2^. As shown by the fit in Figure 3g, this scaling relation is in good agreement with the experimental data for the fitting parameters *d* ≈ 0.77 μm and *A* ∼ 0.155 meV/μm^1/2^. This analysis confirms the square root proportionality of the Rabi splitting to the number of photons for the full set of modes observed in our single-shot experiment, evidencing the utility of the SFA to analyze often-overlooked contributions to light-matter coupling in MDM cavities.

In conclusion, in this work, we used a dye-doped MDM cavity to demonstrate that polaritons generated by the hybridization between excitons and ENZ cavity modes inherit the ENZ character of the cavity mode. We employed two distinct, but complementary, methods to measure the polariton dispersion by varying either the parallel (*k*_||_) or the perpendicular (*k*_⊥_) component of the wavevector in dye-doped MDM cavities.

The first method, based on the angular dispersion of an MDM cavity with fixed thickness, reveals the generation of three polaritonic branches: HP, LP, and MP. As demonstrated by the Hopfield coefficient calculation, HP and LP manifest a marked “cavity-like” behavior with a recognizable ENZ dispersion. In contrast, the third branch (MP) showed an “excitonic” lossy behavior with no ENZ behavior.

The second method is based on the use of the SFA to study the dependence of the strong coupling on *k*_⊥_ by exploiting the curvature of the optical lenses typically used in this setup that create a continuous lateral variation of the cavity thickness. SFA measurements allowed to detect the strong coupling of several consecutive cavity modes (harmonics) with the same exciton transition, within a rapid single-shot measurement. Such analysis revealed an often-overlooked aspect of the collective nature of the strong coupling process, that is the Rabi splitting proportionality to the square root of the number of the involved photons. Our reported Rabi splitting that increases as a function of the lateral position *r*_*q*_ in the sphere-plane geometry is in very good agreement with the theoretical predictions.

Our work opens to polaritonics in the ENZ regime that integrates the highly appealing features of vanishing dielectric permittivity (energy tunneling, phase engineering, slow group velocity, etc.) to the field of strong light–matter interaction. Moreover, our approach proposes the SFA as a new experimental tool to investigate through single-shot measurements continuous variations of the salient parameters involved in the coupling dynamics.

## Methods

### Fabrication and Characterization of ENZ Cavities

The layered metal–dielectric–metal (MDM) structure was fabricated by a multistep process. A 30-nm-thick silver layer was deposited using DC magnetron sputtering in an argon atmosphere (*P* = 4 × 10^–2^ mbar and a deposition power of 20 W). 14 mM R6G in ethanol-PVP solution was spin-coated at 2500 rpm for 1 min on top of the Ag layer to obtain a 315-nm-thick dye-doped PVP layer. The spin-coated sample was annealed for 10 min at 70 °C to ensure an ethanol-free PVP layer. After that, the top 30-nm Ag layer was deposited via DC magnetron sputtering with deposition parameters equal to the aforementioned ones. After deposition of each layer, thickness and optical constant were measured by ellipsometry. *s*- and *p*-polarized reflectance of the ENZ cavities was measured by spectroscopic ellipsometry in an angular range between 25° and 85° with a step of 2°.

### SFA Experiments

A surface force apparatus (SFA) Mark III by Surforce LLC, USA was used in the experiments.^24,30^ One of the Ag-coated cylindrical lenses was fixed on a rigid support, whereas the other one was attached to the free end of a double cantilever spring. The surfaces of the lenses were sufficiently far apart from each other to avoid any mechanical interaction and moved freely in contact with a 40 μL droplet of a PVP-ethanol solution. The droplet was infiltrated between the Ag-coated lenses by capillarity, and it was either doped with a concentration of 1.5 wt % R6G or left undoped.

Transmission spectra were obtained by illuminating the MDM cavity (consisting of the metal layers on the lens surfaces and the dielectric solution) under normal incidence with white light from a halogen lamp. The transmitted light was collected through the entrance slit of an imaging spectrograph (PI Acton Spectra Pro 2300i) aligned with one of the cylindrical lenses, and recorded with a high-sensitivity CCD camera (Andor Newton DU940P-FI). Only a small region of the surface surrounding the contact position was probed, such that *r* ≤ 0.15 mm ≪ *R*, equivalent to a sphere-plane geometry.^31^ A CCD camera image recorded the transmitted intensity *I* as a function of the wavelength λ and position *r* (Figure 3). Multibeam interference created resonance peaks in a spectrogram, i.e., local maxima of the 2*d* intensity function *I*(λ, *r*), corresponding to constructive interference. Spectrograms such as those shown in Figures 3c and 3d were obtained by combining multiple CCD images taken in different but overlapping spectral intervals. Each image was recorded within <1 s, whereas a spectrogram was completed within <20 s.

## Supporting Information Available

Schematic illustration of a metal-dielectric-metal (MDM) resonator; refractive index calculation of PVP-R6G; P-polarization reflectance analysis of the dye-doped MDM cavity; ENZ fit of R6G-doped MDM cavity via ellipsometry measurements and analytical model of the effective dielectric permittivity; three oscillator coupling model and Hopfield coefficients calculation; Gaussian fit of the transmittance spectra acquired via SFA measurements; determination of the number of photons per mode *q* in the cavity (PDF)
