# Supplementary material for: In-Plane and Out-of-Plane Investigation of Resonant Tunneling Polaritons in Metal–Dielectric–Metal Cavities
Source: Nano Lett. 2023 Feb 6;23(4):1489–95. doi: 10.1021/acs.nanolett.2c04864 (PMC9951238; doi:10.1021/acs.nanolett.2c04864)
Supplement: Supplementary file 1 — nl2c04864_si_001.pdf [file nl2c04864_si_001.pdf]

Supporting Information for

In-Plane and Out-of-Plane Investigation of Resonant Tunneling Polaritons in Metal-Dielectric-Metal Cavities

Aniket Patra[†ς], *Vincenzo Caligiuri**[†§], *Bruno Zappone*[§], *Roman Krahne*[ς] *and Antonio De Luca*[†§]

[†] Dipartimento di Fisica, Università della Calabria, via P. Bucci 33b, 87036 Rende (CS), Italy.

[§] Consiglio Nazionale delle Ricerche – Istituto di Nanotecnologia (CNR-Nanotec), via P. Bucci 33c, 87036 Rende, Italy.

[ς] Optoelectronics Research Line, Istituto Italiano di Tecnologia, via Morego 30, 16163 Genova, Italy.

*CORRESPONDING AUTHOR: vincenzo.caligiuri@unical.it, bruno.zappone@cnr.it

SECTION 1 – Schematic illustration of a metal-dielectric-metal (MDM) resonator

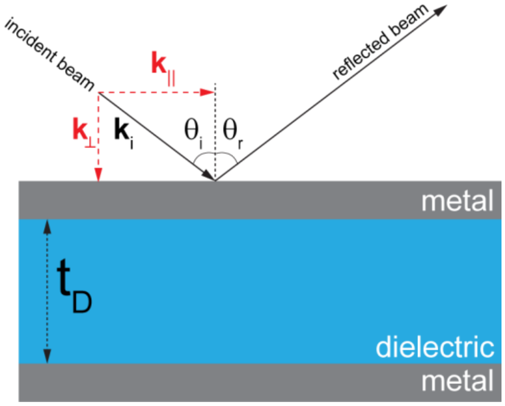

Figure S1. *Schematic illustration of a metal-dielectric-metal (MDM) single-cavity Fabry-Perot resonator.* $\mathbf{k_i}$ *is the incident wavevector with in-plane component* $k_{||}$ *and out-of-plane component* $k_{\perp}$.

SECTION 2 – Refractive index calculation of PVP-R6G

The optical properties of the PVP-R6G layer used in the system described in **Figure 1b** of the main manuscript have been measured *via* spectroscopic ellipsometry.

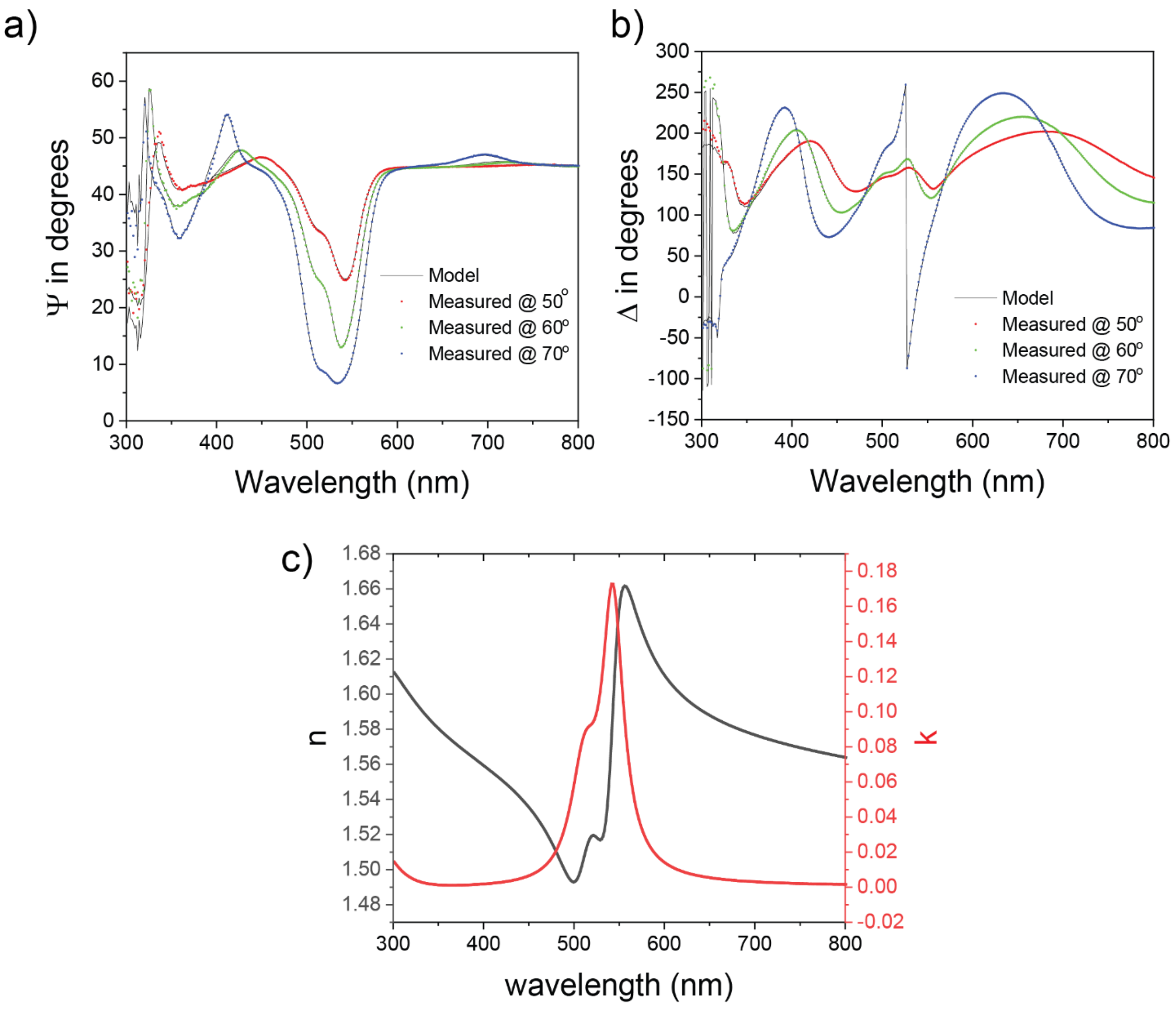

Figure S2*. Measured spectroscopic ellipsometry data and fitting results for PVP-R6G layer on top of a 50nm Ag layer. Figures (a) and (b) show the Ψ and Δ data, respectively, collected at an incident angle of 50º, 60º, and 70º, together with model fits. Figure (c) shows the resulting optical constants for the PVP-R6G layer as calculated by the fitted oscillator model. The index of refraction n is shown as a black line and the extinction coefficient (k) as a red line in figure panel (c).*

The imaginary part of the measured refractive index has been fitted by means of a gaussian function:

$$\sum_{i=1}^{3} A_i e^{-\frac{(x-\mu_i)}{2\sigma_i^2}} \qquad \text{S1}$$

where A_i, μ_i, and σ_i are the amplitude, central wavelength, and linewidth of the ith oscillator, respectively. When the oscillator could be associated with an optical transition, A_i, μ_i, and σ_i corresponded also to the amplitude, wavelength (energy through the relation: E(eV) = 1240 / λ(nm)), and linewidth of the ith optical transition.

	A_i (a. u.)	μ_i (nm)	σ_i (nm)
Oscillator 1	0.0548	302	94.35
Oscillator 2	0.194	508	31.33
Oscillator 3	0.536	546	27.56

The first oscillator does not correspond to any optical transition, but it is the contribution of the PVP polymer matrix. The second oscillator corresponds to the High-Energy (HE) exciton, occurring at 508 nm (2.44 eV). The third one corresponds to the Low-Energy (LE) exciton, occurring at 546 nm (2.27 eV). The amplitude of Oscillator 3 is larger than that of Oscillator 2, confirming the larger transition dipole moment associated with this transition.

SECTION 3 - P-Polarization reflectance analysis of the dye-doped MDM cavity

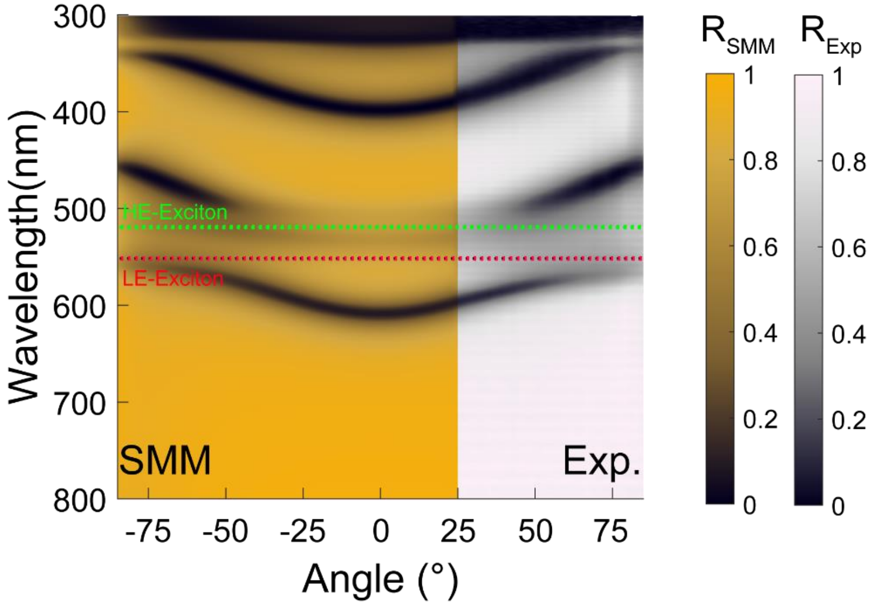

Figure S3*. Experimental and calculated p-polarization reflectance of the doped MDM cavity with R6G embedded in the PVP layer.*

P-polarization angular dispersion of the dye-doped MDM system was measured (gray area) through an ellipsometric setup and numerically calculated (yellow area) *via* Scattering Matrix Method, completing the characterization shown in **Figure 1b** of the main manuscript. The Rabi split was measured for p-polarization both experimentally and numerically.

SECTION 4 - ENZ fit of R6G-doped MDM cavity *via* ellipsometry measurements and analytical model of the effective dielectric permittivity

The effective permittivity ε_{eff} of a R6G-doped MDM cavity was calculated by fitting spectroscopic ellipsometry data with the standard method used for thin multilayers. The calculated real part ε' and imaginary part ε'' of the permittivity were extracted from a multiple Gaussian fit to the measured spectroscopic scan obtained under a 40° angle of incidence and are reported in **Figure 2a**,**b**. The ellipsometry angles ψ and Δ are shown in **Figure S4 a,b** along with the fitted model curve.

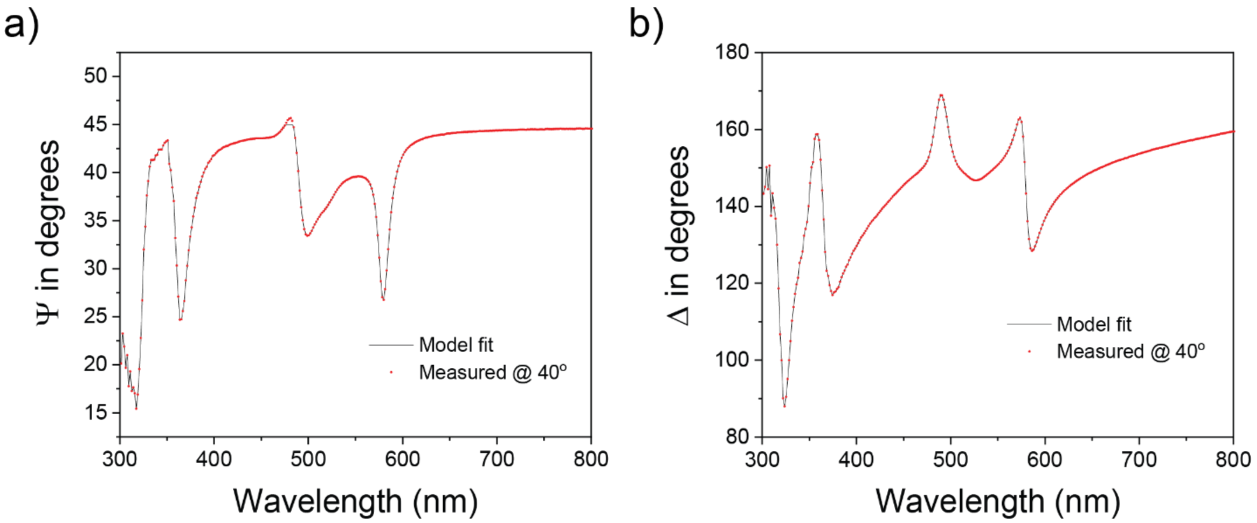

Figure S4. *Measured Spectroscopic ellipsometry data and fitting results for R6G-doped MDM cavity. Figures (a) and (b) show the Ψ and Δ data (red dots) and model fits (solid lines), respectively, collected at an incident angle of 40°.*

The imaginary part ε''was fitted as a sum of five Gaussian oscillators (**eq. S1)** equal to the number of cavity modes. The fitted paraments are given below.

	A_i (a. u.)	μ_i (nm)	σ_i (nm)
Oscillator 1	6.33	301.75	33.24
Oscillator 2	13.00	357.09	6.42
Oscillator 3	48.79	489.19	4.63
Oscillator 4	6.63	513.37	41.33
Oscillator 5	13.85	567.06	17.044

An analytical model for the effective permittivity of a dye-doped MDM cavity can be developed from these ellipsometry results. The full expression of the effective permittivity of the dye-doped MDM system is:

$$\varepsilon_{eff}(\omega) = \varepsilon_{Drude}(\omega) + \sum_{j=1}^{N} \varepsilon_{Lor}(\omega, j) + \varepsilon_{offset} + \varepsilon_{pole}(\omega); \quad \text{S2}$$

Here:

$$\varepsilon_{Drude}(\omega) = \varepsilon_{\infty} - \frac{\omega_P^2}{\omega^2 + i\gamma_D\omega}; \quad \text{S3}$$

is the well-known free-electrons model for metals, which accounts for natural ENZ volume plasmons (i.e., the Ferrel-Berreman mode) and produces accurate results for the Ag layers used in this work.[60] In analogy with pure cavity modes, a Lorentzian oscillator has been assigned to each polaritonic mode. Therefore, the term $\varepsilon_{Lor}(\omega,j)$ represents the jth Lorentzian oscillator associated with the jth polaritonic mode (HP, MP and LP). The expression for $\varepsilon_{Lor}(\omega,j)$ is:

$$\varepsilon_{Lor}(\omega, j) = \frac{A_j\gamma_j\omega}{\omega^2 - \omega_{0,j}^2 - i\gamma_j\omega}; \quad \text{S4}$$

The component ε_{pole} in **Eq. S2** operates only on the real part of the effective permittivity and accounts for out-of-range absorbances:

$$\varepsilon_{pole}(\omega) = \frac{A_P}{\omega_P^2 - \omega^2}; \quad \text{S5}$$

The constant ε_{offset} in **Eq. S2** models the residual polarizability not already accounted for in the ε_{∞} term of the Drude permittivity. All the parameters used in the model are listed in **Table S1**.

Table S1: Values of the parameters used in **Eq. S2** to model the effective permittivity of a dye-doped MDM system,

ε_{Drude}	ε_{∞}	5,75
	ω_P (eV)	9,1
	γ_D (eV)	0,021
ε_{Lor-HP}	A_{HP}	48,01
	γ_{HP} (eV)	0,0235
	ω_{0HP} (eV)	2,535
ε_{Lor-MP}	A_{MP}	7,102
	γ_{MP} (eV)	0,247
	ω_{0MP} (eV)	2,42
ε_{Lor-LP}	A_{LP}	14,18
	γ_{LP} (eV)	0,07
	ω_{0LP} (eV)	2,19
ε_{Pole}	A_p	0,0061
	ω_{pole} (eV)	3,11
ε_{Offset}		2,83

SECTION 5 – Three oscillator coupling model and Hopfield coefficients calculation

The dispersion of the hybridized mode was calculated by solving the eigenvalue problem of the three coupled oscillators. The coupling Hamiltonian is equal to:

$$H = \begin{pmatrix} E_{ph}(\theta) - i\frac{\gamma_{ph}(\theta)}{2} & \frac{\hbar\Omega_{HE/ph}}{2} & \frac{\hbar\Omega_{LE/ph}}{2} \\ \frac{\hbar\Omega_{HE/ph}}{2} & E_{HE} - i\frac{\gamma_{HE}}{2} & \frac{\hbar\Omega_{HE/LE}}{2} \\ \frac{\hbar\Omega_{LE/ph}}{2} & \frac{\hbar\Omega_{LE/HE}}{2} & E_{LE} - i\frac{\gamma_{LE}}{2} \end{pmatrix}$$

E_{ph} is the energy of the undoped MDM cavity, and $E_{HE,}$ and E_{LE} are the energy levels of the high-energy and low-energy exciton of R6G, respectively. $\gamma_{MDM,}$ γ_{HE}, and γ_{LE} are the decay rates of the oscillator, which is also the FWHM (full width at half maximum) of cavity modes and excitons. The terms $\hbar\Omega_{HE/ph}$ and $\hbar\Omega_{LE/ph}$ denote HE exciton-photon and LE exciton-photon coupling strength, and $\hbar\Omega_{HE/LE} = \hbar\Omega_{LE/HE}$ is the interaction potential between the two excitons of R6G, which is considered zero in our case. The parameters used in these models are given below:

E_{ph} and γ_{ph} vary with the angle and their values at the two splitting points are E_{ph} = 2.81 *eV* and E_{ph} =2.08 *eV*, respectively, and γ_{ph} = 0.096 and γ_{ph} = 0.075 *eV*. The other parameters are: E_{HE} = 2.42 *eV*, E_{LE} = 2.28 *eV*, γ_{HE} = 0.174 *eV*, γ_{LE} = 0.112 *eV*, $\hbar\Omega_{HE/ph}$ = 0.169 *eV*, and $\hbar\Omega_{LE/ph}$ = 0.260 *eV*. The full Hamiltonian can, therefore, be simplified as follows:

$$H = \begin{pmatrix} E_{ph}(\theta) - i\frac{\gamma_{ph}(\theta)}{2} & \frac{\hbar\Omega_{HE/ph}}{2} & \frac{\hbar\Omega_{LE/ph}}{2} \\ \frac{\hbar\Omega_{HE/ph}}{2} & E_{HE} - i\frac{\gamma_{HE}}{2} & 0 \\ \frac{\hbar\Omega_{LE/ph}}{2} & 0 & E_{LE} - i\frac{\gamma_{LE}}{2} \end{pmatrix};$$

The complete eigenvalues problem, therefore, reads $HV = \varepsilon V$, where the eigenvalues ε correspond to the energy of the polaritons and can be represented by a diagonal matrix:

$$\varepsilon = \begin{bmatrix} E_{HP} & 0 & 0 \\ 0 & E_{MP} & 0 \\ 0 & 0 & E_{LP} \end{bmatrix};$$

The eigenvectors V correspond to the Hopfield coefficients and can be represented as follows:

$$V = \begin{bmatrix} HP/ph & MP/ph & LP/ph \\ HP/HE & MP/HE & MP/HE \\ HP/LE & MP/LE & MP/LE \end{bmatrix}$$

The Hopfield coefficients, elucidate the mixed nature of a polariton by quantifying the distinct contributions of "matter" and "light".[61–64] In our case, the Hopfield coefficients are three and account for the contribution of the cavity mode, i.e., the photon (ph), the high-energy exciton (HE), and the low-energy exciton (HE) to each of the three polaritons (HP, MP, and LP). The total coefficients are, therefore, nine: LP/ph, MP/ph, and HP/ph (black-triangle-dashed curves in **Fig. S5**) accounting for the cavity (photon) contribution; LP/HE, MP/HE, and HP/HE, accounting for the HE exciton contribution (green-circle-dashed curves in **Fig. S5a-c**); LP/LE, MP/LE, and HP/LE, accounting for the LE exciton contribution (red-square-dashed curves in **Fig. S5a-c**). As the incidence angle is decreased, the interaction between the LP and the cavity mode is gradually reduced, while the interaction between the HP and the cavity mode increases (blue triangle-dashed curves in **Fig. S5a,c**). For small angles, the LP mainly shows a "cavity" nature, while the HP is characterized by a marked "exciton" nature, stemming from the interaction between the cavity mode and the HE exciton. The situation is reversed for angles larger than 50°. The MP, on the other hand, always shows a distinct "exciton" character. At angles smaller than 45°, the MP is mainly determined by the contribution of the LE exciton, while the largest contribution is provided by the HE exciton at larger angles (**Fig. S5b**). In fact, the contribution of the cavity mode to the MP never exceeds 0.2 (**Fig. S5b**).

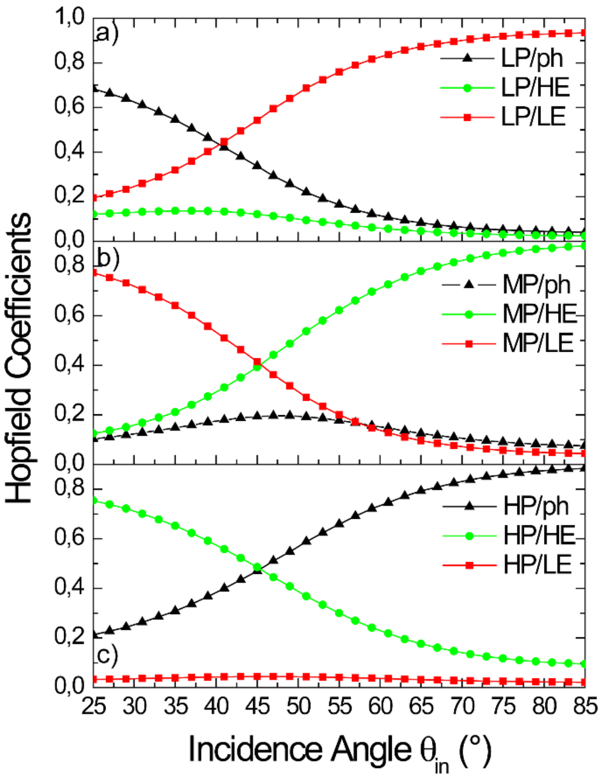

Figure *S5*. Hopfield coefficients calculated for the three polaritonic branches.

For an angle θ_{in} = 40° (**Fig. 2** of the main manuscript**)**, the contribution of the cavity mode to the HP and LP polaritons is significant (~ 0.4). This feature is also reflected by the fact that the HP and LP modes fall in a low-loss spectral region of the PVP-R6G compound with $\boldsymbol{\kappa}$ < 0.02 (see **Fig. S2c**–red curve, calculated by the ellipsometric measurements of Ψ and Δ, as reported in **Fig. S2a-b**). Therefore, the HP and LP branches of the polariton inherit high-quality ENZ features from the cavity mode, and the real part of the effective permittivity crosses the zero line at the polariton energies (or wavelengths in **Fig. 2a**). On the contrary, polaritons belonging to the MP branch always lie within the high-absorbance energy range of R6G. As a consequence, the MP is affected by the lossy features of the R6G absorption transition. This consideration is also supported by the fact that the sum of the Hopfield coefficients inherent to the excitonic contributions (MP/HE and MP/LE) is equal to about 0.82 for θ_{in} = 40°, whereas the MP/ph coefficient is

about 0.18. As a result, the real part of the effective dielectric permittivity does not vanish for the MP branch (**Fig. 2a**).

SECTION 6 - Gaussian fit of the transmittance spectra acquired *via* SFA measurements:

In **Figure S6,** the gaussian fit of the transmittance spectra of the dye-doped MDM cavity measured through the SFA setup is reported. Such a procedure allowed us to precisely determine the spectral position of the oscillators involved in the strong coupling process and to finely calculate the value of the Rabi splitting for each one of the five successive considered harmonics. The involved oscillators correspond to HP, MP, and LP modes reported in the main manuscript, in case of the spectra falling in the high-intensity spectrograph field of view (mode 1, 2, and 3)

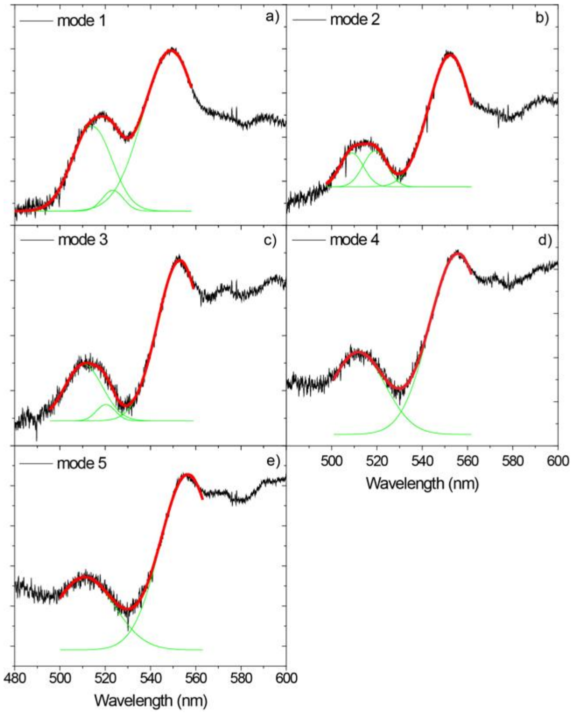

Figure S6. *Gaussian fit of the transmittance spectra measured through the SFA setup for the five consecutive harmonics considered in Figure 3e of the main manuscript.*

In the case of mode 4 and 5, HP branches are barely distinguishable from MP ones. Moreover, since for these modes the HP and MP energies were very close, the fitting procedure converged into only two gaussian oscillators. In the following table, we report the exact value of the central wavelength (and the

conversion in energy units) of the gaussian oscillators used to fit each spectrum of **Fig. S6**, together with the calculated Rabi splitting.

	**Oscillator 1 (nm)**	**Oscillator 1 (eV)**	**Oscillator 2 (nm)**	**Oscillator 2 (eV)**	**Oscillator 3 (nm)**	**Oscillator 3 (eV)**	**Rabi Spliting (eV)**
**mode 1**	514	2.41	519.5	2.387	553	2.242	0.145
**mode 2**	509	2.44	517.5	2.396	553	2.242	0.153
**mode 3**	509	2.44	514	2.412	553	2.242	0.170
**mode 4**	#	#	511	2.424	553	2.242	0.182
**mode 5**	#	#	507.5	2.443	552.7	2.243	0.200

SECTION 7 – Determination of the number of photons *per* mode *q* in the cavity:

To determine the number of photons in a cavity as a function of the cavity thickness, we consider the resonances of a planar MDM cavity (i.e., Fabry-Perot interferometer) excited by a plane wave under normal incidence (**Fig. S7**).

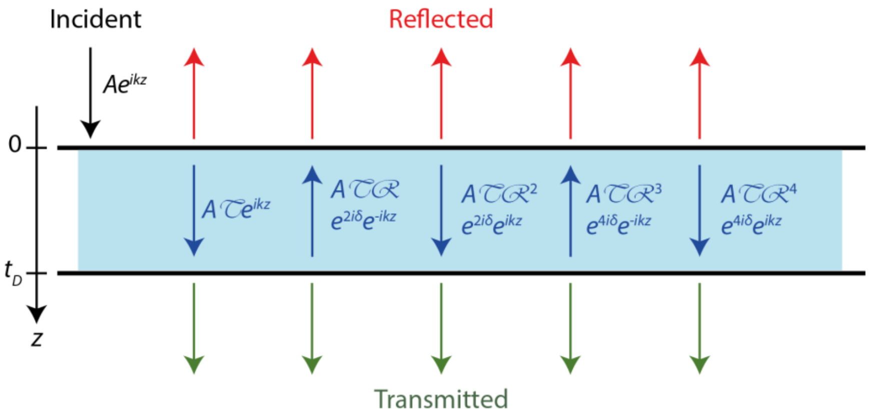

Figure S7. *Multiple-beam reflections within MTM cavity*

We call z the surface normal, t_D the thickness of the central dielectric layer, and $z = 0$ the position of one of the metal-dielectric interfaces. To determine the electric field E in the dielectric layer, we write E as an infinite series of plane waves that propagate forward or backward along z, and are reflected backward or

forward, respectively, by the metal into the dielectric layer. This method of analysis is typically used in the study of thin-film interference, e.g., to determine the properties of Newton rings or calculate the reflectance and transmittance of a Fabry-Perot interferometer [e.g., see Born & Wolf, *Principles of Optics* (1999) Cambridge Press].

The incident wave produces a transmitted wave $A\mathcal{T}e^{ikz}$ propagating along z in the dielectric, where $\mathcal{T}$ is the transmission coefficient, $k = n\omega/c$ is the wavevector, n is the dielectric refractive index, and c is the speed of light (**Fig. S7**). This wave is reflected on the second metal-dielectric interface in $z = t_D$ and produces a second wave $A\mathcal{T}\mathcal{R}e^{2i\delta}e^{-ikz}$ that propagates in the $-z$ direction. The phase delay $\delta = t_D k$ is due to propagation across the thickness t_D, whereas $\mathcal{R} = \rho e^{i\varphi}$ is the reflection coefficient at the metal-dielectric interface. The counter-propagating wave is reflected at the first metal-dielectric interfaces and produces a third wave $A\mathcal{T}\mathcal{R}^{2}e^{2i\delta}e^{-ikz}$ propagating along z. This cycle of forward wave propagation, reflection, backward propagation, and second reflection repeats indefinitely within the dielectric layer to produce the electric field:

$$E = A\mathcal{T}(e^{ikz} + \mathcal{R}e^{2i\delta}e^{-ikz} + \mathcal{R}^2 e^{2i\delta}e^{ikz} + \mathcal{R}^3 e^{4i\delta}e^{-ikz} + \cdots =$$
$$= A\mathcal{T}(e^{ikz} + \mathcal{R}e^{2i\delta}e^{-ikz})\sum_{m=0}^{+\infty}(\mathcal{R}^2 e^{2i\delta})^m = A\mathcal{T}e^{ikz}(1 + \mathcal{R}e^{-2i(kz-\delta)})/(1 - \mathcal{R}^2 e^{2i\delta}) \qquad \text{S6}$$

The intensity $I = |E|^2$ of the electric field is:

$$\frac{I}{I_a} = \mathcal{T}^2 \frac{1+\rho^2+2\rho\cos(2kz-2\delta-\varphi)}{1+\rho^4-2\rho^2\cos(2\delta+2\varphi)} \qquad \text{S7}$$

where $I_a = |A|^2$ is the incident wave intensity. The electromagnetic energy $\mathcal{E}$ is proportional to $\int I dz$, where the integral is calculated from $z = 0$ to $z = t_D$. Integration of **Eq. S7** gives:

$$U = I_a \mathcal{T}^2 t_D f(2\delta) \qquad \text{S8}$$

where we have introduced the function:

$$f = \frac{1+\rho^2+2\rho\,[\sin(2\delta+\varphi)-\sin\,(\varphi)]/2\delta}{1+\rho^4-2\rho^2\cos(2\delta+2\varphi)} \qquad \text{S9}$$

In view of our SFA experiments on strong cavity-exciton coupling, we focus on the exciton wavelength λ_E, so that $U = (I_a\mathcal{T}^2/k_E)\delta_E f(2\delta_E)$ depends on t_D only through the variable $\delta_E = t_D k_E$. The function f is periodic in δ_E with period π and the energy U has maxima for $\delta_E = \delta_q = q'\pi$, where $q' = q - \varphi/\pi$ and q is an integer (**Fig. S7**). These maxima correspond to cavity resonances, i.e., maxima of transmittance, occurring at increasing cavity thicknesses t_q for an increasing resonance order q. At resonance, the function f becomes:

$$f_q = \frac{1+\rho^2}{(1-\rho^2)^2}\left[1-\frac{2\rho}{(1+\rho^2)^2}\frac{\sin\varphi}{q'\pi}\right] \qquad \text{S10}$$

For the Ag layers considered in our experiments, $\rho < 1$ and $\varphi \approx \pi$. Therefore, the second term in the bracket of **Eq. S10** is much smaller than one, particularly for large orders q, so the maximum f_q is practically independent of q and slightly larger than one. As a consequence, the energy at a resonance is $U_q = (I_a \mathcal{T}^2/k_E)\delta_q f_q \approx (I_a \mathcal{T}^2/k_E)\delta_q$, *i.e.*, it is directly proportional to the cavity thickness t_q (**Fig. S7**). Moreover, U only depends on $\delta_E = k_E t_D$ and the peak width σ_δ as a function of δ does not depend on q.

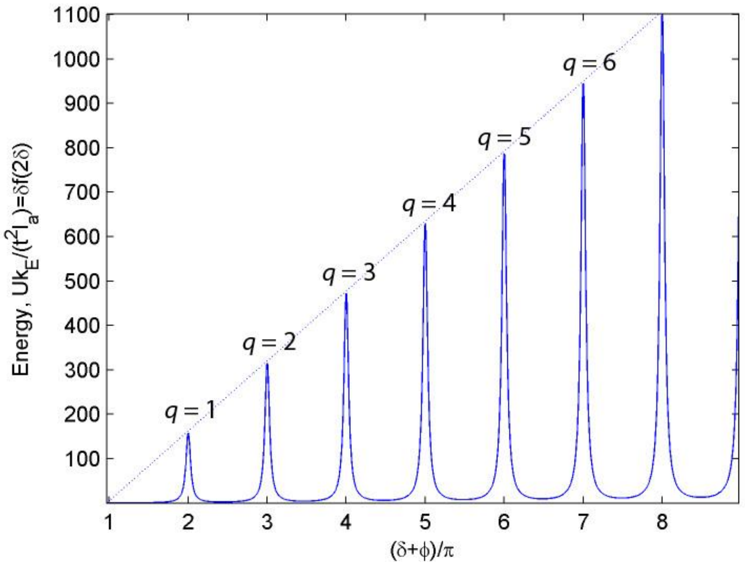

Figure S7. *Energy U of the electromagnetic field in an MDM cavity as a function of $\delta = t_D k_E$, where t_D is the cavity thickness, and k_E is the exciton wavevector. The resonance of order q is obtained for $\delta_q = t_q k_E = q\pi - \varphi$, where φ is the phase shift by reflection on the metal layer. The dotted line shows that the resonance value U_q increases linearly with q.*

The mode energy U_q is proportional to the number of photons m_q, namely $U_q = m_q \hbar\omega_E$, where $\hbar\omega_E$ is the photon energy. Because U_q is directly proportional to t_q, so is the number of photons m_q.
